# The evolution of disease criteria in multiple sclerosis: underlying motives and broader implications

**DOI:** 10.1186/s12883-026-04641-x

**Published:** 2026-01-23

**Authors:** Kristofoor E. Leeuwenberg, Lennart H. van der Molen, Marianne Boenink, Edo Richard

**Affiliations:** 1 Department of Neurology, Donders Institute for Brain, Cognition and Behaviour, Radboud Research Institute for Medical Innovation, Nijmegen, The Netherlands; 2 Department of IQ healthcare, Ethics of healthcare, Radboud University Medical Center, Nijmegen, The Netherlands; 3 Department of Public & Occupational Health, Amsterdam University Medical Center, University of Amsterdam, Amsterdam, The Netherlands

**Keywords:** Multiple sclerosis, Diagnosis, Biomarkers, Magnetic resonance imaging

## Abstract

**Introduction:**

Disease criteria for multiple sclerosis (MS) have changed significantly over the past three decades. The development of biomarkers, particularly MRI, has played an essential role in this process. These changes can lead to several broader implications, which are not always intended or recognized. However, they can have a major impact on patients, healthcare and society. We investigated the changing disease criteria, explored the underlying motives and discuss their implications.

**Methods:**

We identified all publications on MS diagnostic criteria between 1983 and 2024. Through conventional content analysis, we systematically examined the aims of each revision and identified the underlying problems and factors motivating these aims. Furthermore, we assessed the changes made to the content in each revision.

**Results:**

Since the transition from the Poser research criteria to the McDonald clinical criteria in 2001, there has been a significant change in the aims of the criteria, which has continued in subsequent McDonald revisions. Two explicit new aims were to integrate MRI into the diagnostic scheme and to reach an earlier diagnosis of MS whenever possible. Furthermore, the emphasis moved towards criteria that are easy to apply in everyday clinical practice, rather than being exclusively for research purposes. This shift is at least partially connected to the emergence of disease-modifying treatments, which have introduced new diagnostic and monitoring needs. Additionally, advancements in MRI research have contributed to this process. As a result, MRI has become central to the diagnostic criteria over the years, while clinical symptoms now play a much smaller role.

**Discussion:**

The shift in the criteria has several broader implications. It has a major impact on how MS is conceptualized as a disease and has expanded the disease spectrum to include new terms such as radiologically isolated syndrome (RIS). It also influences our understanding of the natural course of the disease and calls for a careful balance between early diagnosis and misdiagnosis. Awareness of the process of changes and their broader consequences can help balance the benefits and potential negative effects and helps critically frame the decisions of the 2024 revisions.

**Supplementary Information:**

The online version contains supplementary material available at 10.1186/s12883-026-04641-x.

## Introduction

In the last three decades diagnosis and treatment of multiple sclerosis (MS) have changed significantly. The diagnostic criteria have been repeatedly adjusted from being primarily based on clinical symptoms to incorporation of imaging and other biomarkers, with MRI currently being the cornerstone of diagnosis [1, 2]. A noticeable shift in the criteria is the increased importance of biomarkers, which are defined as measurable indicators of underlying biological or pathogenic processes [3]. Biomarkers have broadened the spectrum of MS and introduced new concepts, including clinically isolated syndrome (CIS) and radiologically isolated syndrome (RIS) [4]. These categories include individuals who would have previously been considered healthy, and show how the emphasis on biomarkers has shifted our understanding of MS.

Similar changes in disease criteria have occurred in other neurological diseases, including Alzheimer’s Disease and more recently also Parkinson’s Disease [5, 6]. This process, in which biomarkers are increasingly proposed as a solution for addressing biomedical and health issues, is referred to as ‘biomarkerization’ [7, 8]. It is associated with presumed benefits for patients. For example, it is considered a way to improve diagnostic specificity and detect early and pre-symptomatic disease stages, creating opportunities for early therapeutic intervention [5]. However, it may also have broader implications for the way we conceptualize disease, for how disease is experienced, and for the health care system or society as a whole. Such implications may not always be beneficial. A diagnosis of a chronic neurological condition, for example, has far-reaching consequences, and treatments always involve risks that should be carefully evaluated.

The research criteria introduced by Poser in 1983 were the first to include laboratory and paraclinical evidence, such as MRI, in the diagnostic scheme [2]. Before 1983, for more than a century there were only a handful of diagnostic schemes available for MS, which were all based on purely clinical features and focused on selecting patients for therapeutic trials, with the criteria of Schumacher (1965) being most widely recognized [9, 10]. Here we reconstruct and investigate the evolution of diagnostic criteria for MS from 1983 to the present. We focus on relapsing-remitting MS, the most common form of MS. Our aim is to identify the underlying motives and rationale that contributed to shifts in the criteria. Furthermore, we explore how the content of the criteria itself has changed over time and discuss the broader implications of these shifts. Analyzing this process helps to determine whether the revisions have met their intended purposes and may help prevent unintended negative consequences in future revisions. As criteria are updated periodically, this analysis helps critically frame the decisions of the 2024 revisions, which have recently been published [11].

## Methods

The manuscripts of published MS diagnostic criteria (1983–2024) were identified through PubMed, using combinations of the search terms ‘diagnosis’, ‘diagnostic’, ‘criteria‘ and ‘MS/multiple sclerosis‘. These manuscripts were systematically analyzed using conventional content analysis in two steps. First, we identified the perceived problems, aims and the motivations underlying these aims [12]. The aims were identified by analyzing common phrasing, such as ‘we aim to …’ or ‘there is a need for ...’. These sections were highlighted and categorized by topic, and re-identified through subsequent revisions of the criteria. Perceived problems were identified in the text by either reviewing the considerations related to stated aims or by noting any explicitly mentioned problems.

Additionally, we examined changes in the content of the criteria over time. Along with analyzing the original criteria manuscripts to examine the changes in the role of different criteria components, we reviewed commentaries on these publications to explore how they were received in the MS field. These commentaries were obtained by searching PubMed using combinations of the terms ‘diagnosis,’ ‘diagnostic criteria,’ and ‘commentary,’ ‘critique,’ ‘limitations,’ or their synonyms, supplemented by references cited in the retrieved articles.

## Results

Five sets of diagnostic criteria for MS have been published between 1983 and 2024. The Poser criteria were developed by 27 experts from Canada, the USA, and the UK^2^. Subsequent revisions in 2001, 2005, 2010, and 2017 were led by Ian McDonald and an expanding international panel [1, 13–15]. The aims and problems of each criteria are outlined below (Table 1; Supplementary Table 1).

**Table 1:** Summary of the main aims and problems statements for each criteria revision, extracted from the text of the original criteria publications, including key changes that were made to the content of the diagnostic criteria

Criteria version	Intended use	Aims	Problems	Key criteria changes
Poser 1983^2^	Research	- construct exacter criteria for research purposes - attain high specificity to exclude patients with mimicking diseases such as ADEM - include ancillary procedures into the diagnostic scheme - criteria should not overrule the clinical judgment of a neurologist	- subjective judgement in existing diagnostic schemes - discrepant terminology in what is considered probable and definite MS - difficulty differentiating MS from mimicking diseases such as ADEM	- construction of new criteria, diagnosis based on clinical attacks proving dissemination in time and space - in absence of sufficient clinical evidence, clinical signs combined with CSF abnormalities can reach a laboratory-supported diagnosis - MRI and other ancillary studies are considered ‘paraclinical evidence’ and serve as an extension of the clinical neurological exam
McDonald 2001^13^	Clinical	- construct criteria suitable for clinical application - integrate MRI into the diagnostic scheme - clarify and simplify criteria where possible - construct clear criteria for primary progressive disease - reach an earlier and more objective diagnosis of MS (implicit)	- previous definitions too subjective and not clear enough in the context of treatment decisions - subcategories (clinically definite and laboratory supported) not contributive - risk for misdiagnosis remains problematic - accessibility of MRI may be limited in some countries - new scheme not validated for diverse populations - imaging may reveal ‘silent disease’	- detailed MRI criteria to demonstrate dissemination in time and/or space as alternative for a second clinical attack were added - the distinction between clinical and laboratory-supported subgroups was abandoned - diagnosis still based on clinical attacks proving dissemination in time and space - CSF may be helpful to confirm dissemination in time if there is insufficient clinical or MRI evidence to prove this
McDonald 2005^14^	Clinical	- continuation of the aims set in 2001; reaching an earlier diagnosis now as explicit aim - incorporate new evidence where available - clarify role of spinal cord imaging in criteria	- MRI criteria in previous scheme too stringent - previous criteria for spinal cord were deemed confusing and unclear - lower sensitivity and specificity of criteria in populations beyond white European and North American adults continues to be problematic	- further definition of the use of spinal cord imaging - minor adjustments to MRI criteria to make them less stringent
McDonald 2010^15^	Clinical	- continuation of the aims set in 2001 and 2005 - reliable use of MS criteria across diverse populations - limit the number of necessary MRI scans for diagnosis where possible - harmonize MRI criteria between subtypes	- further elaboration on previously stated problems in 2001 and 2005 - updated criteria may change some of the outcomes of natural history studies	- dissemination in time can now be demonstrated with a single MRI showing both enhancing and non-enhancing lesions. - the MRI criteria for dissemination in space were made more flexible
McDonald 2017^1^	Clinical	- continuation of the aims set in 2001, 2005 and 2010 - emphasize the importance of appropriate application of criteria to prevent misdiagnosis - emphasize the important role of MRI for diagnosis (implicit)	- further elaboration on previously identified problems in 2001, 2005 and 2010 - an increasingly strong focus on timely diagnosis might increase risk of misdiagnosis - there is still a lack of a biomarker to differentiate between MS phenotypes or monitor CNS damage - management of RIS remains unclear and should be high-priority area for further research	- cortical MRI lesions are included as an extra option to prove dissemination in space - the presence of positive oligoclonal bands in CSF is regarded as sufficient proof of dissemination in time on its own - further modification of MRI criteria, making them more flexible and easier to meet

Abbreviations: *MS* Multiple Sclerosis, *ADEM* Acute Disseminated Encephalomyelitis, *MRI* Magnetic Resonance Imaging, *CSF* Cerebrospinal Fluid, *CNS* Central Nervous System

### Poser criteria aims

The primary aim of the new Poser criteria was to establish a more precise definition of MS for the selection of patients for therapeutic trials, as well as for epidemiological surveys, evaluation of new diagnostic procedures, and assessment of disease activity. Poser’s panel identified three key problems that motivated this aim: (1) the terminology used for definite MS at the time was considered inconsistent, (2) the subjectivity of judgments in the existing diagnostic schemes, and (3) the challenges of differentiating MS from mimicking diseases, such as acute disseminated encephalomyelitis (ADEM). All three issues were deemed problematic for including patients in multicenter trials. The new criteria were intended for research purposes and not for clinical application. An explicit aim was to avoid overruling the clinical judgment of a neurologist when diagnosing MS in a patient who did not fully meet the criteria.

### McDonald criteria aims

The introduction of the McDonald 2001 criteria marked a clear shift in aims that were largely maintained and further elaborated in subsequent McDonald criteria. These will be discussed individually for each aim below.

The primary aim of the McDonald criteria was to develop diagnostic criteria suitable for clinical application, rather than only research purposes. This broadening of the scope was motivated by the emergence of therapies and new implications of a diagnosis for treatment decisions. The McDonald panel stated that Poser’s criteria relied too heavily on subjective judgment to be used for this new clinical purpose. To attend to this primary aim several sub-aims were established, including (1) the aim to clarify definitions that are considered confusing and (2) the aim to simplify the diagnostic criteria where possible. The latter includes reducing the number of necessary MRI scans for a diagnosis where possible. These two sub-aims were responses to feedback from the MS medical community, which deemed several definitions and aspects of the MRI criteria in the earlier revisions (2001, 2005) as unclear and confusing. A third sub-aim was the reliable application of the criteria for populations beyond white European and American adults. This was stated as a problem in the early criteria (2001, 2005) and was subsequently addressed in 2010 and 2017 when more evidence was available.

Another explicit aim of the McDonald criteria was to integrate MRI into the diagnostic scheme. This was motivated by the need for a more objective diagnosis in the context of treatment decisions. A second motivation for this aim was new available evidence from MRI studies showing a high sensitivity while maintaining specificity for the diagnosis, with each revision incorporating additional evidence [16, 17]. Most of these MRI studies were supported by MAGNIMS, a large independent international research collaboration founded in 1990.

From 2005 onwards enabling an early diagnosis of MS became an additional aim. This was motivated by the increasing evidence that early initiation of treatment led to positive treatment outcomes. Moreover, early diagnosis was said to alleviate the uncertainty experienced by the patient. However, the 2017 revision pointed out that it might also increase the risk of misdiagnosis, noting that the benefits of early diagnosis must be carefully balanced with the risks of incorrect diagnosis.

### Content of criteria

**Fig. 1:**
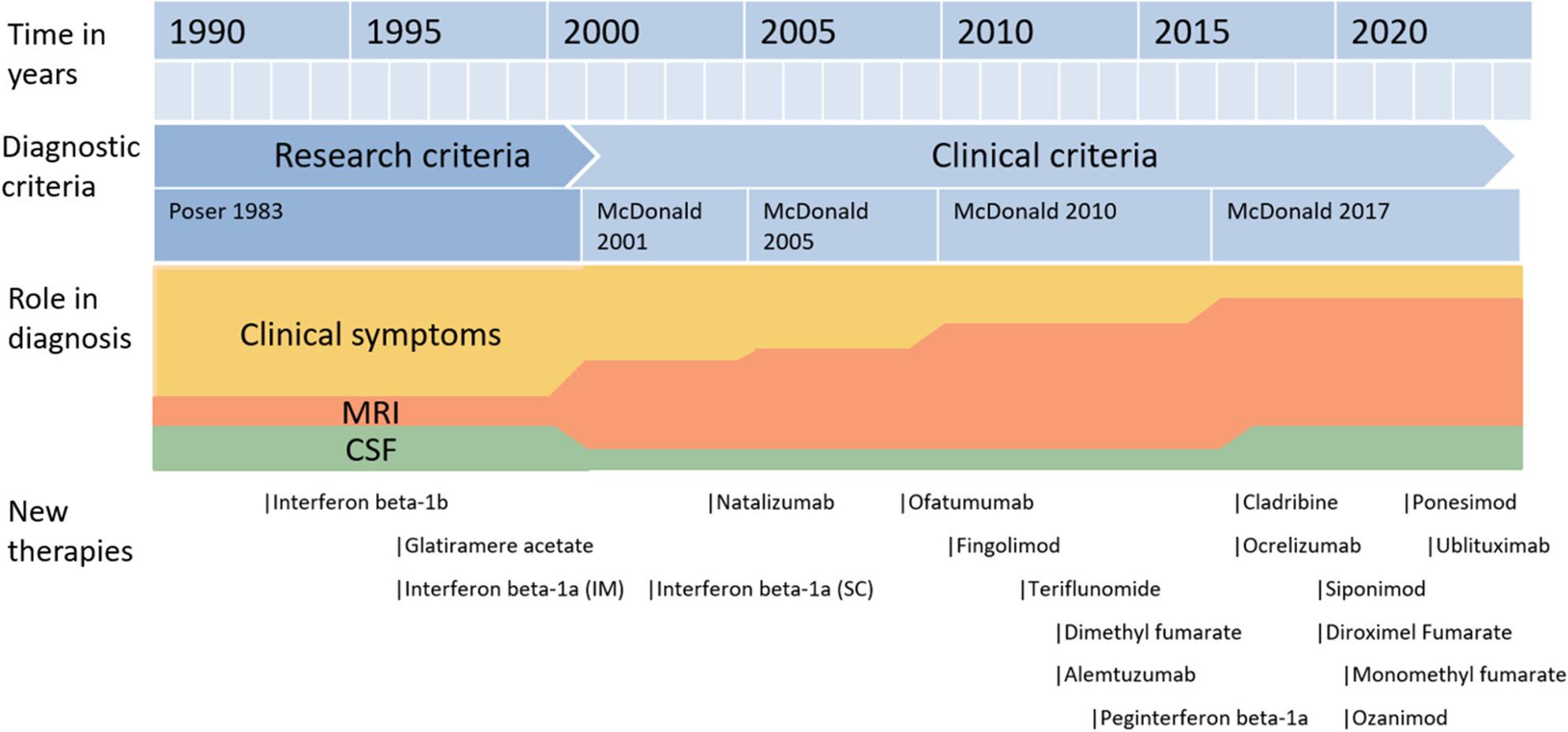
Overview of evolution of diagnostic criteria and treatment of MS (1990–2024). The role in diagnosis is an illustration, and is not based on quantitative analysis. Therapy timeline was determined by their initial approval from either the EMA (European Medicines Agency) or FDA (Food and Drug Administration). MRI: magnetic resonance imaging, CSF: cerebrospinal fluid, IM: intramuscular, SC: subcutaneous.

The criteria have evolved over time in response to stated aims and perceived issues. While the core principle of dissemination in time and space (originated in 1954 [18] and articulated in Poser criteria) remains, the roles of clinical symptoms, MRI, and cerebrospinal fluid (CSF) oligoclonal bands have shifted (Fig. 1). The next section outlines these changes.

#### Clinical symptoms

The definition of clinical symptoms in the criteria has remained relatively consistent over the years in terms of function, but clinical symptoms have become less important due to the increasing role of other components. In Poser’s criteria, clinical symptoms played a central role: a definitive diagnosis could be made based solely on history and clinical examination, termed ‘clinically definite MS.’ Paraclinical evidence of MS lesions, such as evoked potentials, CT, and MRI scans, was seen as an extension of the neurological exam. When clinical evidence was insufficient, laboratory tests (CSF analysis) could help establish a ‘laboratory-supported’ diagnosis. This set of technologies was described as a way to expand research inclusion, but Poser’s panel allowed for researchers to restrict inclusion to clinically diagnosed patients. This implies that the clinically definite group was prioritized. In the 2001 McDonald criteria the subgroups ‘clinically definite’ and ‘laboratory-supported’ were removed. While the description and use of clinical symptoms were largely retained, the definition of ‘clinical symptoms’ became stricter. A clinical attack now required objective evidence from neurological examination or other methods, and symptoms based only on patient history were no longer accepted. This strictness was upheld in later revisions, with a minor modification in 2010: in the ‘appropriate context,’ historical reports of typical MS symptoms were considered valid evidence, provided that at least one attack was confirmed by neurological examination findings or paraclinical evidence for a definitive diagnosis.

#### MRI

Over the years, MRI has shifted from a minor to the key element in the diagnostic process. In Poser’s criteria, the role of MRI was described as part of the ‘paraclinical evidence’ group and could serve as an extension of neurological examination. MRI was scarcely mentioned beyond this. The role of the MRI scan grew significantly with the introduction of the 2001 McDonald criteria. MRI was now considered capable of confirming both dissemination in time (DIT) and space (DIS), replacing the need for a second clinical attack in some cases. The MRI criteria incorporated four elements: the number of lesions, their anatomical locations, whether a lesion shows contrast enhancement, and whether new lesions appeared between two distinct MRI scans [16, 17]. In subsequent revisions (2005, 2010, 2017), the MRI criteria were further developed, making them easier to meet and apply in practice, thereby enhancing their role in the diagnostic process. While MRI initially focused on the brain, spinal cord lesions could replace brain lesions—a concept further expanded in the 2005 McDonald revision. In 2010, a significant step was taken, as DIT could now be demonstrated with a single MRI (when both enhancing and non-enhancing lesions were present), making MRI practically equivalent to a second clinical attack. In 2017, MRI thresholds for DIT and DIS were further reduced, adding cortical lesions alongside juxtacortical lesions as valid evidence. MRI’s diagnostic and monitoring role was emphasized, and the panel advised that brain MRI be obtained for all patients being considered for MS. For individuals with MRI lesions without MS-symptoms (‘radiologically isolated syndrome’), the consensus was to continue to require a clinical attack for diagnosis of MS.

#### Cerebrospinal fluid and other biomarkers

Over the years, CSF has maintained a largely unchanged function, serving mostly as a supportive element, with a minor re-evaluation in the 2017 revision. In Poser’s criteria, CSF played a slightly larger role, as it was a key component of the ‘laboratory-supported’ classification. This category was removed in the 2001 McDonald criteria, and CSF analysis subsequently served primarily as additional evidence for DIT when clinical or MRI features were insufficient. In the 2017 revision, its role was slightly increased, as it is now considered sufficient evidence for DIT on its own.

Other biomarkers have had a limited role in the diagnostic criteria. In Poser’s criteria, various tests, including CT, evoked potentials, neuropsychological assessments, and urological tests, were listed under ‘paraclinical evidence’ alongside MRI. However, these were not expanded upon and were excluded in later McDonald criteria. In the 2010 and 2017 revisions, it was noted that neuromyelitis optica spectrum disorders are considered distinct from MS and may be further differentiated through serological testing for aquaporin-4 (AQP4) and myelin oligodendrocyte glycoprotein (MOG) antibodies.

## Discussion

Over the last three decades, the MS criteria have undergone considerable changes, with a clear shift towards the use of biomarkers, especially MRI, to identify MS lesions. We will further discuss the motives and implications below.

### Motivations

The first key motivation behind the changes in criteria was the introduction of treatment options in 1995, which led to a focus on easily applicable clinical criteria and an early diagnosis. A diagnosis began to carry more consequences, making an uncertain diagnosis problematic. The first McDonald criteria in 2001 responded to this by extending the scope of the criteria from research to clinical practice. Over the years treatment options expanded rapidly, and evidence showed a better effect if therapy is started early in the disease process (the so called ‘window of opportunity’)[19]. This was seen as a strong incentive for an early diagnosis, with every successive criteria revision after 2001 becoming less stringent to allow for easier application. Filippi et al. showed the 2017 McDonald criteria reduce the time to diagnosis by 4.6 years compared to purely clinical criteria, and 10 months compared to the 2010 criteria [20].

Another important motive for the shift towards a more central role for biomarkers was the new available evidence from MRI studies supported by MAGNIMS. Key contributions were the MRI criteria of Barkhof et al. (1997) and Tintore et al. (2000), with subsequent studies further developing these criteria over the years [16, 17]. Alongside these developments, members of the MAGNIMS consortium began to merge with the McDonald diagnostic criteria panel since 2005. This integration is noteworthy, as there is a clear connection between MAGNIMS and the content adjustments in more recent revisions. An example of this connection is that in 2016, new MRI criteria were proposed by researchers on behalf of MAGNIMS, who were also involved in the 2017 criteria revision [21]. On one hand, involving experts of the MRI technique in the diagnostic criteria panel is advantageous and valuable for the advancement of the diagnostic criteria. On the other hand, however, the participating members are evaluating their own work, which could result in potential conflicts of interest and perhaps heightened expectations regarding the role of MRI.

### Consequences

The changes in criteria, notably the expanded role of MRI, has brought about a range of consequences, both intended and unintended (Table 2). We will discuss these implications in more detail below.

**Table 2:** Summary of ongoing shifts in MS criteria and their potential broader implications

Shifts	Potential broader implications
More biological and biomarker-centered diagnosis	- Change in the conceptualization of MS as a disease - Increased incidence of ‘silent disease’ (radiologically isolated syndrome), causing previously healthy individuals to be classified as ill - Risk of contradictory biomarker findings, with paradoxical increase of diagnostic (and prognostic) uncertainty for patients - Restriction of wide applicability outside specialized centers due to the need for advanced diagnostic technology - Risk of reduced attention to patients’ symptom experiences in clinical practice
Strive for earlier diagnosis	- Increased risk of misdiagnosis, resulting in inappropriate and potentially harmful treatment - Bias in knowledge of natural course of the disease (Will Rogers phenomenon), complicating comparisons of incidence rates, prognosis assessment, and trial outcomes.

Abbreviations: *MS* Multiple Sclerosis

#### Conceptualization

An important consequence of the expanded role of MRI is its influence on how MS is perceived, shifting the focus towards a more biological rather than clinical definition. Where MRI was initially used primarily for diagnosis, it has since been utilized for monitoring and assessing disease activity as well. Other examples of this expansion include the introduction of additional parameters, such as cortical lesions, and the incorporation of new imaging regions, like the spinal cord [1, 14]. These radiological findings are strongly considered a representation of the ‘real’ disease process (the pathology), with clinical symptoms being a less sensitive downstream phenomenon. This shift towards a more biological view is visible in the later McDonald criteria’s statement that ‘silent disease’ on MRI is allowed to replace a second clinical attack for diagnosis. It is also reflected in the way a purely clinical diagnosis is portrayed in the papers: In Poser’s criteria it is well accepted, while in McDonald’s 2005 criteria it is deemed ‘appropriate when MRI and other paraclinical examinations are not possible’ and in the 2017 criteria it is advised to obtain an MRI in alle patients being considered for a diagnosis of MS. It is important to note that this shift carries the risk of clinical symptoms receiving less attention, a change that may extend into clinical practice. As patient symptoms become less relevant to the doctor, they may be given less attention in the communication, which can have consequences for the way care is given to and experienced by the patient [22].

#### Knowledge of natural course

Another consequence of the expanding role of MRI in the recent MS-criteria is a shift in our understanding of the condition’s natural course [23]. In response to the 2010 criteria, it was commented that simplifying the MRI criteria could lead to more patients being diagnosed with MS, who would otherwise have been classified as having clinically isolated syndrome [24]. This implies that the MS population defined by the latest McDonald criteria will encompass individuals at an earlier stage of the disease or with less active disease in general, leading to higher incidence rates and a more favorable overall natural course compared to previous definitions of MS. This concept of shifts in incidence and prognosis due to evolving disease definitions is referred to as the Will Rogers phenomenon, and in the case of MS is strongly linked to the increased sensitivity of MRI imaging [25–27]. Consequently, caution should be exercised when assessing prognosis for a patient, or when comparing incidence rates or trial results [28, 29]. It is worth noting that one of the key objectives of the Poser criteria in 1983 was to enable the effective comparison of study populations, but that the evolution of the new criteria over time has ultimately complicated this objective.

#### Early diagnosis and misdiagnosis

Since MS criteria are not validated against a pathological gold standard, their sensitivity and specificity are generally evaluated on their ability to predict a second clinical attack that confirms clinically definite MS in CIS patients [20, 30]. Thus, sensitivity reflects the inclusion of CIS patients who will develop MS, while specificity relates to exclusion of CIS patients who will not ultimately develop MS. Early diagnosis has been a clear aim since the 2005 McDonald criteria, further emphasized in subsequent revisions, and has resulted in greater focus on sensitivity rather than specificity in new criteria [20, 30]. Additionally, revisions were largely validated in populations with high likelihood of MS and positive predictive value may be lower in more general populations, especially when MRI criteria are not appropriately applied [1, 31]. Together, these create a conflict between the focus on early diagnosis and a substantial risk of misdiagnosis, which can result in serious harm [32]. Misdiagnosis (an incorrect MS diagnosis) is very common and may occur in approximately 15% of cases [33]. A study in 2016 showed that 70% of patients who had been misdiagnosed with MS had received one or more disease-modifying treatments, and one third had received such therapies for 10 years or longer. One third of these patients had experienced at least one morbidity as a result of their misdiagnosis, including death in one patient with fulminant neuromyelitis optica (0.9%) [34]. In this study, 60% of misdiagnoses were partly attributed to physicians placing too much emphasis on MRI abnormalities in patients with nonspecific neurological symptoms. While antibody testing has improved differentiation from other demyelinating diseases, conditions like migraine and cerebrovascular disease remain among the most commonly confused with MS [33]. These conditions generally present with distinct clinical features but may be incorrectly classified as CIS. When nonspecific white matter lesions are present on MRI and fulfill the MS diagnostic criteria for DIS, this leads to diagnostic confusion [35]. Although the criteria have addressed the classification of CIS over the years, applying them in clinical practice remains a common challenge. As emphasized in the recent revision [11], the exclusion of other conditions remains a crucial component of the diagnostic criteria.

#### Radiologically isolated syndrome

A fourth important consequence related to an increasingly more MRI-based disease definition are incidental radiological findings suggestive of MS in patients not suspected for a diagnosis of MS. The term ‘silent disease’ was first used for this concept in the 2001 McDonald criteria and later replaced by ‘radiologically isolated syndrome’ (RIS) in 2009, and used in the 2010 and 2017 criteria [1, 13, 15, 36]. RIS prevalence ranges from 0.06% to 0.7% in the general population [37], but in some populations where atypical MRI abnormalities are being identified as RIS more rapidly it can be as high as 3.7% [38]. Although addressed only to a limited extent in the MS criteria papers, dedicated diagnostic criteria for RIS have been formulated [39] and RIS has become a common issue in daily clinical practice. Only one third of individuals with RIS are diagnosed with MS within 5 years, and the uncertain meaning of RIS may have a considerable impact on the patient’s quality of life [40]. A long-term cohort study revealed that half of the participants remained asymptomatic for at least 10 years following the identification of RIS [41]. Furthermore, autopsy series have described incidental pathological findings associated with MS in cases without MS-related symptoms, suggesting that symptoms may remain absent throughout one’s life [42]. With regard to this, it is important to recognize the impact of the terminology used to assess these incidental findings. Although RIS is more neutral than the earlier term ‘silent disease’, it is still strongly associated with MS. The question remains if RIS should be considered an early phase of MS disease or seen as an independent entity. Labelling it as RIS places it within the MS disease spectrum, even though many individuals with RIS do not develop symptoms over time. Current European guidelines offer limited guidance for management of RIS [43-47]. The latest Dutch guideline does not recommend starting treatment in individuals with RIS, but this is an active topic of discussion as recent trials suggest it may delay the onset of symptoms [48-51]. These studies demonstrated that treatment in individuals with RIS extended the time before clinical conversion or new MRI lesions appeared. Since a single exacerbation may carry the risk of incomplete recovery, this approach can potentially offer clinical benefit. Some members of the 2017 McDonald panel argued that individuals with RIS often already experience nonspecific symptoms related to MS disease, such as fatigue or cognitive impairment, and that diagnosis and early treatment should not be postponed further. However, a proposal to allow diagnosis of MS in individuals with RIS and presence of CSF oligoclonal bands did not receive general support in the 2017 panel as the risk of misdiagnosis was considered too high.

#### 2024 revisions of the McDonald criteria

In 2023, a new committee of 56 international experts convened to update the criteria, with the stated aim of making it easier to diagnose MS more quickly and accurately. The subsequent revisions to the criteria have been published very recently [11]. Several new biomarkers have been added to the criteria, including the optic nerve as fifth localization for DIS, kappa free light chains as a biomarker in CSF, and the central vein sign and paramagnetic rim lesions as new MS markers on MRI. Furthermore, the presence of MRI abnormalities is considered a necessity for diagnosis of MS [52]].

One explicit aim of the new revisions is to move towards a more biological diagnosis, as reflected by the revision to allow for a diagnosis of MS in a subpopulation of individuals with RIS. This change marks a significant step, drawing parallels with other neurological diseases: a more biological conceptualization of the disease and increasing drive for early diagnosis has led to definitions for asymptomatic disease in Alzheimer’s and Parkinson’s disease [5, 55]. It is important to recognize that this step may have a profound effect on the conceptualization of the disease, as well as experiences of the individuals concerned. Given these developments, it’s important to emphasize that before the introduction of MRI these individuals were considered healthy, and half of them remain symptom-free after 10 years.

#### Strengths and limitations

Our comprehensive analysis, which includes the aims and motivations of the criteria alongside their content, provides the necessary perspective to understand the shift towards a biologically defined disease. This may help clinicians apply the criteria responsibly in daily practice. The use of content analysis is suitable for this approach but also has limitations. Although it is a recognized qualitative research method [12], a certain degree of interpretive subjectivity may be involved. For instance, this may include determining what qualifies as an aim or a problem within the text, as well as organizing these elements into overarching themes.

## Conclusion

The diagnostic criteria for MS have changed considerably over the last decades, with an increasingly important role for biomarkers, and specifically MRI. These changes are motivated by the emergence of MS therapies as well as significant developments in the research field of MRI imaging. Biomarkers have provided more insight in the way the disease manifests in the central nervous system and help to diagnose MS at an earlier disease stage. However, this shift also had broader implications, including an increasingly biological conceptualization of MS and the rise of incidental imaging findings. The latter means that individuals who were once deemed healthy are now classified as patients, and the management strategy for this group remains a matter of debate.

The 2024 revisions to the MS criteria further advance the transition towards a more biological and biomarker-based diagnosis [11]. This final adoption follows the consistent direction set in 2001 and even marks a considerable acceleration. In light of this, a critical assessment of the role of biomarkers and their potential broader negative implications during the years following this recent adoption is crucial to ensure that unintended harmful consequences are not overlooked.

## Supplementary Information

Supplementary Material 1

## Data Availability

The data used in this study are based on publicly available literature.

## Abbreviations

ADEM: Acute Disseminated Encephalomyelitis
AQP4: Aquaporin–4
CIS: Clinically Isolated Syndrome
CNS: Central Nervous System
CSF: Cerebrospinal Fluid
CT: Computed Tomography
DIT: Dissemination in Time
DIS: Dissemination in Space
EMA: European Medicines Agency
FDA: Food and Drug Administration
MAGNIMS: ‘Magnetic Resonance Imaging in MS’ (international research collaboration)
MOG: Myelin Oligodendrocyte Glycoprotein
MRI: Magnetic Resonance Imaging
MS: Multiple Sclerosis
RIS: Radiologically Isolated Syndrome
UK: United Kingdom
USA: United States of America
