# Supplementary material for: The evolution of disease criteria in multiple sclerosis: underlying motives and broader implications
Source: BMC Neurol. 2026 Jan 23;26:103. doi: 10.1186/s12883-026-04641-x (PMC12896151; doi:10.1186/s12883-026-04641-x)
Supplement: Supplementary file 1 — Supplementary Material 1 [file 12883_2026_4641_MOESM1_ESM.docx]

Supplementary Table 1. Complete content analysis of aims (red) and problems (blue) including references to the texts from which they were derived.

|  | **Aim/problem** | **Reference in text** |
| --- | --- | --- |
| **Poser 1983** | Aim 1: more exact criteria for conducting research, evaluating new diagnostic procedures and estimating disease activity | ‘Today there is … process in MS’ (p. 227) ‘The main reason … with definite MS’ (p. 230) |
|  | Aim 2: construct criteria that are specific enough to exclude patients with mimicking diseases such as ADEM | ‘The need to … have definite MS’ (p. 230) |
|  | Aim 3: criteria are meant for research and should not overrule the clinical judgment of a neurologist | ‘The guidelines may …diagnosis of MS’ (p. 230) |
|  | Aim 4: include ancillary procedures into the diagnostics scheme | ‘The Schumacher criteria … more up-to-date guidelines’ (p. 231) |
|  | Problem 1: discrepant terminology in what is considered probable and definite MS | ‘One problem with … ‘definite’ in another’ (p. 227) |
|  | Problem 2: subjective judgement in current diagnostic schemes | ‘Another problem is … and neurophysiological procedures’ (p. 227) |
|  | Problem 3: difficulty differentiation MS from mimicking diseases such as ADEM | ‘A major concern … multiple separate lesions’ (p. 230) |
|  | Problem 4: ongoing diagnostic uncertainty for the patient | ‘To a patient … will be worthwhile’ (p. 230) |
| **McDonald 2001** | Aim 5: create diagnostic criteria that can be used by a practicing physician, and that are also suitable for clinical trials | ‘The panel set … for clinical trials’ (p. 121) |
|  | Aim 6: to integrate MRI into the diagnostic scheme (due to its unique sensitivity to pathological change) | ‘The panel also … criteria for MS’ (p. 121) |
|  | Aim 7: to construct a scheme for primary progressive disease | ‘The panel also … criteria for MS’ (p. 121) |
|  | Aim 8: clarify definitions of MS | ‘The panel also … classification and descriptions’ (p. 121) |
|  | Aim 9: simplify diagnostic criteria where possible | ‘The panel also … classification and descriptions’ (p. 121) |
|  | Aim 10: retain as many useful features from previous criteria as possible | ‘While refining the … of existing criteria’ (p. 121) |
|  | Aim 11 (implicit): more objective diagnosis of MS | ‘Obtaining objective evidence … time and space’ (p. 122)  ‘Requiring objective clinical … MS for treatment’ (p. 125) |
|  | Aim 12 (implicit): early diagnosis of MS | *Not mentioned in 2001 manuscript but referred to in McDonald 2005 manuscript*: ‘the intent of … false-positive diagnosis’ (p. 840) |
|  | Problem 5 (implicit): previous definitions unclear | ‘The Panel also … classifications and descriptions’ (p. 121) |
|  | Problem 6: current definitions for clinical evidence are still too subjective for accurate diagnosis of MS and needs to be more objective because its implications for treatment | ‘Obtaining objective evidence … time and space’ (p. 122)  ‘Requiring objective clinical … MS for treatment’ (p. 125) |
|  | Problem 7: subcategories for MS (clinically definite / laboratory supported) are not contributive and therefore unnecessary | ‘Subcategories that define … are unnecessary’ (p. 122) |
|  | Problem 8 (implicit): there is a risk for misdiagnosis, and therefore there must be *no better explanation* for the clinical/paraclinical abnormalities | ‘The panel emphasizes … to be made’ (p. 125) |
|  | Problem 9: accessibility of advanced technologies (MRI) as well as the quality of these tests may be limited in other countries | ‘However, the panel … analysis vary worldwide’ (p. 125) |
|  | Problem 10: current new scheme based on data from adults (10-59) | ‘The panel’s recommendations … cannot be overemphasized’ (p. 125) |
|  | Problem 11: risk for misdiagnosis because of other diseases mimicking MS | ‘Several MS-like … an MS diagnosis’ (p. 126) |
|  | Problem 12: imaging may reveal ‘silent disease’ | ‘Imaging undertaken for … may be desirable’ (p. 126) |
| **McDonald 2005** | Aim 2, 5, 6, 7, 8, 9, 12 | ‘The intent of … utility and validity’ (p. 840) ‘The goals of … difficult to implement’ (p. 841) ‘Establishing a diagnosis … to be problematic’ (p. 844) ‘The 2005 revisions … for the diagnosis’ (p. 844) |
|  | Aim 13: incorporate new evidence where available | ‘The intent of … utility and validity’ (p. 840) |
|  | Aim 14 (implicit): clarify role of spinal cord imaging in criteria | ‘It was recommended … spinal cord lesions’ (p. 843) |
|  | Problem 13: potential lower sensitivity and specificity of criteria in populations beyond white European and North American adults | ‘Other studies and … be optimally useful’ (p. 840) ‘The criteria have … Western adult populations’ (p. 841) |
|  | Problem 14: previous criteria considered unclear and confusing | ‘Other studies and … be optimally useful’ (p. 840) |
|  | Problem 15: the criteria may be less adequate in a general neurology practice setting | ‘It is less … a different condition’ (p. 841) |
|  | Problem 16: the criteria may be less adequate to identify an alternative diagnosis in a patients with CIS fulfilling the criteria for MS (lack of specificity) | ‘It is less … a different condition’ (p. 841) |
|  | Problem 17): incorrect interpretation of previous criteria emerged that diagnosis mainly relies on MRI, while clinical symptoms are still very important | ‘The McDonald Criteria … diagnosis of MS’ (p. 841) |
|  | Problem 18): previous criteria for objective clinical evidence may have been too strict | ‘The original McDonald … an MS diagnosis’ (p. 841) |
|  | Problem 19): MRI criteria in previous scheme too stringent | ‘The MRI requirements … time and space’ (p. 842) |
|  | Problem 20): previous criteria spinal cord deemed confusing and unclear | ‘It was recommended … the diagnostic workup’ (p. 843) |
|  | Problem 21): diagnosis of primary progressive MS continues to be problematic | ‘Establishing a diagnosis … to be problematic’ (p. 844) |
| **McDonald 2010** | Aim 9, 12 | ‘These revisions simplify … and widespread use’ (p. 292) ‘Such changes are … required MRI examinations’ (p. 293) |
|  | Aim 15): more widespread use of MS criteria across populations | ‘These revisions simplify … and widespread use’ (p. 292) ‘Since the revision … Criteria were derived’ (p. 292) |
|  | Aim 16: limit the amount of necessary MRI scans for diagnosis | ‘Such changes are … required MRI examinations’ (p. 293) |
|  | Aim 17: harmonize MRI criteria between subtypes where possible | ‘To harmonize MRI … criterion for DIS’ (p. 295) |
|  | Problem 3, 12, 13, 15 | ‘The panel concluded … neurology practice populations’ (p. 293) ‘In applying the … considered and excluded’ (p. 293) ‘The McDonald Criteria … and Latin Americans’ (p. 295) ‘As a consequence … consistent with MS’ (p. 298) ‘A proportion of … fact have MS’ (p. 298) |
|  | Problem 22: updated criteria may change some of the outcomes of natural history studies | ‘The panel acknowledges … what different criteria’ (p. 298) |
| **McDonald 2017** | Aim 3, 5, 8, 9, 12, 13, 14 | ‘The increasing incorporation … more specific diagnosis’ (p. 162) ‘New data, emerging … and their usefulness’ (p. 162) ‘Challenges in making … diagnose multiple sclerosis’ (p. 162) ‘Rather, the 2017 … frequency of misdiagnosis’ (p. 162, 164) ‘The 2017 McDonald …and clinical practice’ (p. 170) |
|  | Aim 17: emphasize the importance of appropriate application of McDonald Criteria to prevent misdiagnosis | ‘Misdiagnosis had harmful … the McDonald criteria’ (p. 164) |
|  | Aim 18 (implicit): emphasize the important role of MRI for diagnosis | ‘The panel recommended … cost, or contra-indication’ (p. 166) |
|  | Problem 11, 12, 13, 14 | ‘Challenges in making … diagnose multiple sclerosis’ (p. 162) ‘With increasing availability … radiologically isolated syndrome’ (p. 168) |
|  | Problem 23: timely diagnosis might increase risk of misdiagnosis | ‘The increasingly strong … risk of misdiagnosis’ (p. 164) |
|  | Problem 24: lack of a biomarker to differentiate between MS phenotypes or monitor disease activity | ‘Other diagnostic biomarkers … for future research’ (p. 170) |

Table abbreviations:
MS: Multiple Sclerosis
ADEM: Acute Disseminated Encephalomyelitis
MRI: Magnetic Resonance Imaging
CIS: Clinically Isolated Syndrome
DIS: Dissemination in Space
